# Permanent Interstitial Cesium-131 Brachytherapy in Treating High-Risk Recurrent Head and Neck Cancer: A Prospective Pilot Study

**DOI:** 10.3389/fonc.2021.639480

**Published:** 2021-03-18

**Authors:** Michael Kharouta, Chad Zender, Tarun Podder, Rod Rezaee, Pierre Lavertu, Nicole Fowler, Jason Thuener, Shawn Li, Kate Clancy, Zhengzheng Xu, Min Yao

**Affiliations:** ^1^ Department of Radiation Oncology, University Hospitals Seidman Cancer Center, Case Western Reserve University, Cleveland, OH, United States; ^2^ Department of Otolaryngology Head and Neck Surgery, University of Cincinnati School of Medicine, Cincinnati, OH, United States; ^3^ Department of Otolaryngology Head and Neck Surgery, University Hospitals Seidman Cancer Center, Case Western Reserve University, Cleveland, OH, United States

**Keywords:** brachytherapy, cesium 131, recurrence, head and neck, re-irradiation

## Abstract

**Purpose/Objectives:**

To establish the feasibility and safety of intraoperative placement of cesium-131 (Cs-131) seeds for re-irradiation in recurrent head and neck cancer (HNC).

**Methods:**

Patients with resectable recurrent HNC who were deemed to have a high risk of second recurrence were eligible. Immediately after tumor extirpation, seeds were implanted in the surgical bed based on the preoperative treatment plan with intraoperative adjustment. The surgical bed and the seeds were covered with a regional flap or microvascular free flap. A CT of the neck was obtained on postoperative day 1 for evaluation of the postoperative dose distribution. Patients were followed 1 and 3 months after surgery, then every 3 months in the first 2 years.

**Results:**

From November 2016 to September 2018, 15 patients were recruited and 12 patients received treatment per protocol. For the patients who had implants, the sites of initial recurrence included 10 neck alone, 1 neck and larynx, and 1 neck/peristomal. The median follow-up was 21.4 months. After surgery, patients remained hospitalized for a median of 6 days. There were no high-grade toxicities except two patients with wound complications requiring wound care. Eight patients had recurrences, three locoregional alone, three distant alone, and two with both locoregional and distant recurrences. Only one patient had an in-field failure. Five patients died, with 1- and 2-year overall survival of 75 and 58%.

**Conclusions:**

Cs-131 implant after surgical resection in recurrent HNC is feasible and safe. There were no unexpected severe toxicities. Most failures were out-of-field or distant.

**Clinical Trial Registration:**

ClinicalTrials.gov, identifier NCT02794675.

## Introduction

Head and neck cancers (HNC) represented an estimated 53,260 new diagnoses of malignancy in the United States in 2020, with 10,750 estimated deaths (1). Most patients present with loco-regionally advanced disease. Radiation is a principal treatment modality in HNC, either as definitive therapy or after surgery, and often administered with concurrent platinum-based chemotherapy. With modern multimodality therapy, overall survival for these patients continues to improve and typically exceeds 40–70% in modern series (2–4). Locoregional recurrence rates remain high and are the common mode of failure, with tumors of oropharyngeal origin and HPV-related having a relatively better prognosis (2, 5).

Locoregional recurrence in HNC can be particularly morbid, and survival rates at 1 and 2 years following recurrence are poor (5). The primary treatment for recurrence of HNC is surgical resection if possible, often followed by adjuvant radiotherapy to the resection bed, especially for patients with high-risk features (3, 4). Both of these treatments can be complicated by prior radiotherapy. Adjuvant radiotherapy after resection for recurrence can be challenging, as many organs at risk (OAR) near the primary site received significant dose from the primary course of radiation. Severe late toxicity is not uncommon even with the use of intensity-modulated radiation therapy (IMRT) (6). Depending on the site of recurrence, these toxicities may be prohibitive to re-irradiation with external beam radiotherapy (EBRT) techniques, particularly when considered in conjunction with the increased risk of surgical complications such as wound dehiscence, tissue necrosis, or carotid blowout (6–8). In a meta-analysis of re-irradiation for recurrent or second primary HNC, 28% of over 3,700 patients across 39 studies underwent postoperative re-irradiation. Their rates of grade 3+ acute and late toxicities were 32 and 29% respectively, with radionecrosis, dysphagia, and trismus among the most common grade 3–4 late toxicities (7).

Several recently reported attempts to explore newer modes of re-irradiation have focused on highly conformal external beam treatments, including stereotactic body radiation therapy (SBRT) and proton therapy, with some SBRT series showing local control and toxicity comparable to IMRT and other conventional conformal techniques (9). However, brachytherapy is a particularly intriguing modality for this purpose as it attempts to theoretically deliver maximal dose conformality to spare unwanted dose to nearby normal tissues, and allows a sufficiently high re-irradiation dose to the post-operative bed for control of microscopic disease. Brachytherapy can be performed at the time of surgery following primary resection, which eliminates treatment delay for the patient.

The use of Cesium-131 (Cs-131) implants is shown to be feasible in the postoperative setting for recurrent HNC in several small series (8, 9). The relative dosimetric properties of Cs-131 compared to iridium-192 or Iodine-125 isotopes, which include a lower mean energy and short half-life at 9.7 days, have made it an excellent candidate for treatment not only in recurrent HNC, but in the treatment of recurrent brain tumors, inoperable non-small cell lung cancer, and recurrent pelvic malignancies (10–13). Due to the favorable properties of both brachytherapy and Cs-131, we sought to explore the feasibility of Cs-131 implants in recurrent HNC after surgery at our institution. We report our preliminary experience using Cs-131 seed implantation as adjuvant treatment for patients with recurrence of their HNC who undergo salvage surgery.

## Materials and Methods

This is a prospective clinical trial approved by our institutional Internal Review Board (IRB) and is partly supported by IsoRay™ (Richland, WA). The aims of this study were to assess the feasibility and safety associated with Cs-131 brachytherapy in patients with recurrent head and neck cancer undergoing salvage surgery to ensure that any morbidity does not overshadow any measurable oncologic cure.

### Eligibility Criteria and Patient Selection

Eligible patients were age 18–90 years with Karnofsky performance status >60, with resectable, recurrent HNC after previous radiotherapy. All patients were reviewed in multidisciplinary tumor board and deemed to be at high-risk for second failure due to recurrent disease adjacent to critical structures such as the carotid artery, skull base, deep cervical musculature, or other areas that would limit the possibility of en-bloc resection, and were thus deemed candidates for post-operative radiotherapy regardless of primary site. Patients with active pharyngocutaneous fistula, exposed carotid artery preoperatively requiring sacrifice or bypass intra-operatively, or distant metastasis (except for a single lung nodule/2^nd^ lung primary) or HIV-positivity were not eligible. All patients signed IRB-approved informed consent.

### Seed Description and Pre-Planning Procedures

**Figure 1:**
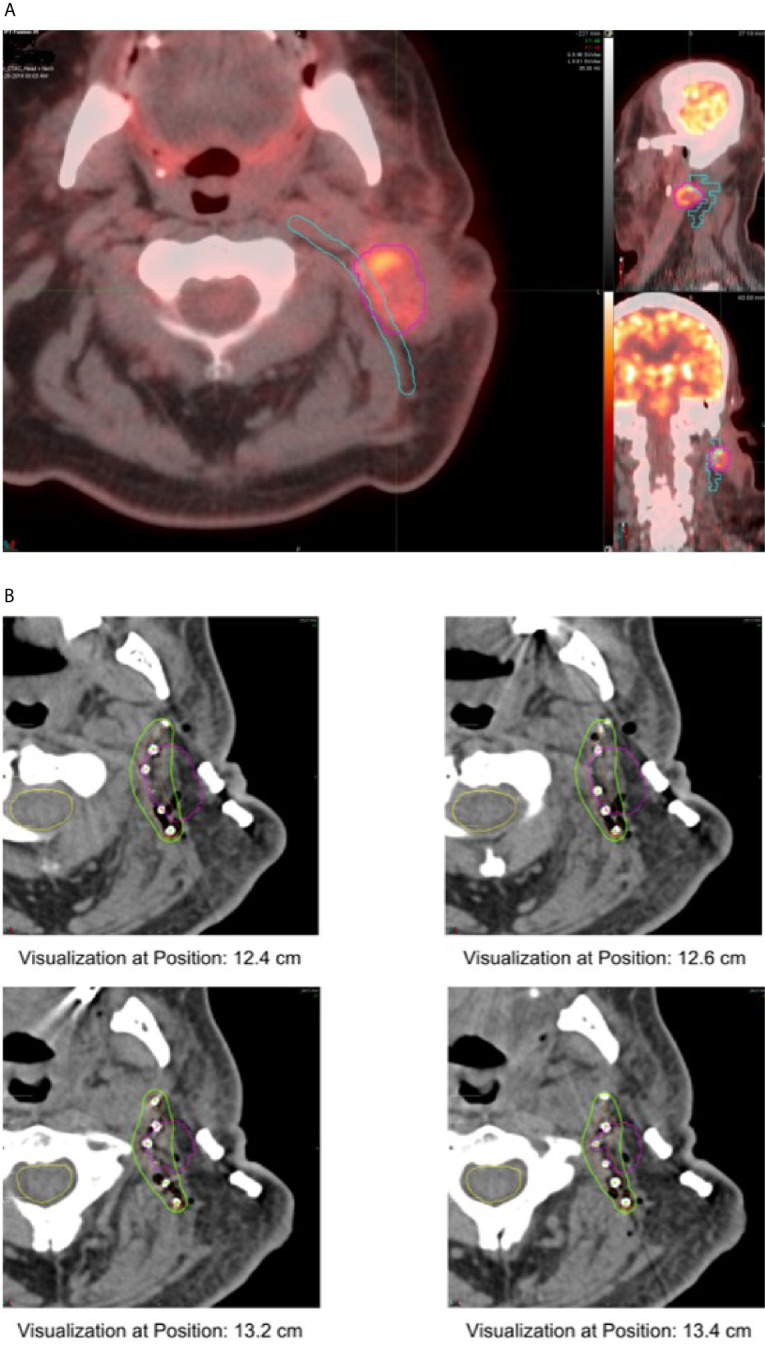
**(A)** Pre-operative plan showing Cs-131 strand implant location along predicted tumor bed. **(B)** Post-operative plan showing implanted Cs-131 seeds on diagnostic CT, pre-operative tumor contour in purple.

Cs-131 seeds were provided by IsoRay™ (Richland, WA). Seeds were supplied in a mesh or strand configuration. The seeds are encased in a 0.05 mm titanium shell, and contain radioactive Cs-131 isotope surrounding a 4 mm gold marker. The strength and number of the Cs-131 seeds were estimated based on a preoperative treatment plan using diagnostic computed tomography (CT) images, as well as positron emission tomography (PET) imaging. The geometry and size of the resection cavity was estimated by the radiation oncologist and head and neck surgeon, and a target volume was delineated on the CT images (**Figure 1A**). Using MIM Symphony LDR™ treatment planning software, version 6.5 (Cleveland, OH, USA), the seeds were placed in a single, optimal plane with 1 cm seed-to-seed spacing to cover the estimated resection cavity. The seed air kerma strength was iteratively adjusted in the planning software, such that a dose of 60–70 Gy was delivered to a prescription point located 5 mm perpendicular to the center of the implant plane. The treatment plan was reviewed by the medical physicist, radiation oncologist, and head and neck surgeon. The prescription dose was adjusted based on the previous radiation dose received and the dose to the spinal cord. The composite dose (dose previously received plus the implant dose) was kept ≤ 140 Gy. Therefore, for patients with recurrence after definitive radiation, the implant dose was between 60 and 65 Gy. For patients recurred after postoperative radiation, the implant dose was between 65 and 70 Gy. The composite dose to the spinal cord was limited to ≤ 50 Gy. A custom mesh and/or set of strands with pre-specified seed spacing was then ordered. In most cases, especially those with larger uncertainty in the size or geometry of the estimated resection cavity, an extra strand of seeds was ordered and used as needed. An identical set of dummy seed mesh/strands was ordered for facilitation of intraoperative adjustment to avoid unnecessary radiation exposure.

In cases where the seeds would be in proximity to critical vascular structures or the mucosal/cutaneous surface a vascularized pedicle flap, free tissue transfer was planned. A thin adipofacial anterolateral thigh free flap (2–3mm thick) was used most frequently for vascular coverage and additional fat/muscle could be harvested and contoured to prevent seed extrusion through mucosal or cutaneous interfaces. Additional soft tissue overlying the seeds lowered radiation exposure and helped to achieve a dose rate limit that was acceptable (< 6 mR/h at 1 meter distance) for patient discharge.

### Intraoperative Planning and Postoperative Dose Verification

**Figure 2:**
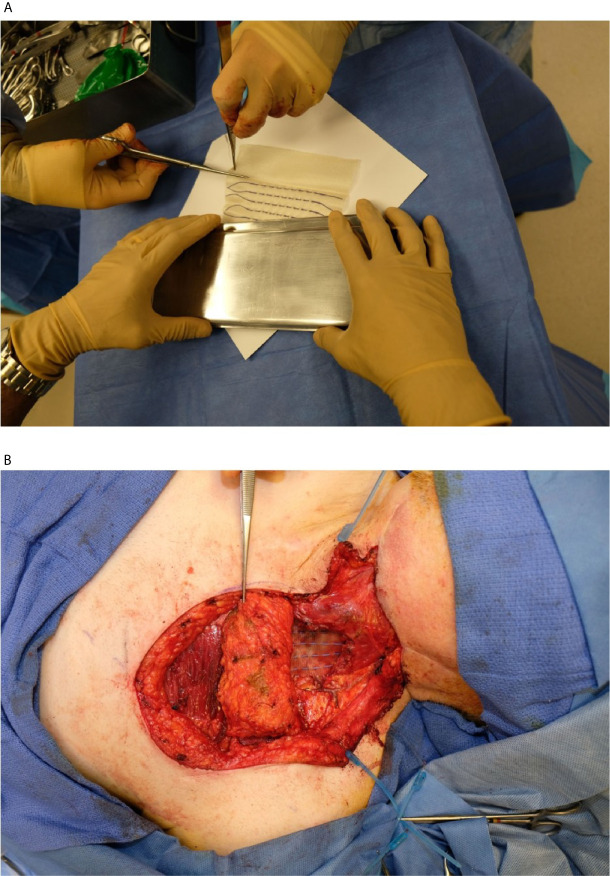
**(A)** Cs-131 strands being removed from sterile packaging for implant. Each strand contained a custom number of seeds spaced at 1 cm apart. **(B)** Exposed resection cavity with several Cs-131 strands implanted per the pre-operative plan.

Immediately after tumor extirpation, the seeds were implanted in the surgical bed based on the preoperative treatment plan with intraoperative adjustment. The mesh containing seeds was secured by suture. The surgical bed and seeds were covered with a regional flap or microvascular free flap (**Figure 2**). Radiation exposure was measured immediately post-surgery, on day 1 post-surgery, and on 5–8 days post-surgery. A CT scan of the neck was obtained on postoperative day 1 for postoperative treatment planning to confirm the dose distribution of the implant (**Figure 1B**).

### Follow-Up and Statistical Analysis

Patients were seen for follow-up at 1 and 3 months after surgery, then every 3 months in the first 2 years and every 6 months thereafter. Toxicities and disease status were recorded prospectively at every follow-up. A CT of the neck was obtained 1–2 months after surgery and a PET/CT was obtained about 3 months after surgery.

Survival estimates were performed using the Kaplan-Meier method. Overall survival was determined from the date of brachytherapy implant to patient death or last follow-up. Progression-free survival was determined from the date of brachytherapy to any progression of disease (either by imaging or pathologic diagnosis) or death. Locoregional failure-free survival was determined from the date of brachytherapy to any progression of disease at the primary site or regional lymphatics. An in-field recurrence was defined as recurrent gross tumor in contact with regions receiving 100% or greater of the implant prescription dose. Distant metastatic failure-free survival was determined from the date of brachytherapy to any occurrence of metastatic disease.

## Results

### Patient Characteristics and Treatment

From November 2016 to September 2018, 15 patients were recruited, and 12 patients received seed implantation. Of the patients who did not receive protocol treatment, one had disease progression before surgery and went to hospice, and two were determined to be low-risk intraoperatively after tumor resection.

Patient characteristics and treatment parameters for the 12 patients who had seed implants are summarized in **Table 1**. There were 11 male and 1 female, with a median age of 75 (52-86) years. Primary sites at initial diagnosis included five oropharynx, three larynx, three skin, and one oral cavity. Recurrent sites of disease included 10 neck alone, 1 both neck and larynx, and 1 neck/peristomal. The interval between recurrence and previous radiation ranged from 3.7 to 103.8 months, with a median of 21.9 months. The interval between previous radiation and Cesium implantation was 4.2–105.1 months, with a median of 22.7 months.

**Table 1:** Patient characteristics and treatment.

**Median age (years)**	75 (52–86)
**Gender**	
Male	11
Female	1
**Site of initial diagnosis**	
Oropharynx	5
Larynx	3
Skin	3
Oral cavity	1
**Site of recurrence**	
Neck alone	10
Neck and larynx	1
Neck/peristoma	1
**Time from RT To recurrence (months)**	21.9 (3.7–103.8)
**Time from RT To implant (months)**	22.7 (4.2–105.1)
**Previous RT dose (Gy)**	70 (50–74)
**Dose implanted (Gy)**	65 (60–70)
**Total cumulative dose (Gy)**	130 (120–140)
**Total seed implanted**	35 (11–68)
**Total seed activity (mCi)**	101.2 (38.8–182.2)

The median radiation dose from the initial course of radiation treatment was 70 Gy (range 50–74 Gy). Cs-131 implant dose ranged from 60 to 70 Gy (median 65 Gy) depending on the previous dose the patient had received and the dose to the critical structures, mainly the spinal cord. The median total cumulative dose was 130.1 Gy (range 120–140 Gy). Total implanted seeds ranged from 11 to 68 (median 35). The median seed activity was 2.8 mCi (range 2.5–3.5 mCi) and total seed activity ranged from 38.8–182.2 mCi (median 101.2 mCi).

### Radiation Safety

Radiation exposure rate was measured using a Victoreen 451B Fluke ion chamber survey meter at 1 meter from the implanted site immediately post-procedure, on day 1 post-procedure, and on 5–8 days post-procedure before patient discharge. Forty two measurements were taken from 12 patients ranging from 2.0 to 6.6 mR/h immediately post-procedure, 1.1–4.7 mR/h on post-operative day 1, and 0.7–2.7 mR/h on post-operative day 5–8. Per NRC regulations using an occupancy factor of 0.25, the calculated dose rate limit for patient release was < 6.0 mR/hr. All patients were below this threshold on 1 day post-procedure and the exposure rate for all patient was < 2mR/h at discharge from hospital.

### Post-Implant Quality Assurance

In order to examine the post-implant movement of the seeds and effect on the dose distribution, CT images obtained at follow-up visits, including the CT as part of the PET/CT, were rigidly registered to the postoperative day 1 CT for the initial seven patients using MIM treatment planning software. The DICOM coordinates of each seed were obtained to determine their movement. The average observed seed movement was found to consistently increase with every subsequent CT acquired. By 60 days after implantation when the implanted Cs-131 seeds deposited 99% of the prescribed dose, the average deviation was 4.3 mm and mean bulk displacement of the entire mesh implant was 2.5 mm. Kaplan-Meier plots obtained for the probability of a seed not having been observed to move a given distance revealed that after 60 days, 98.8% of the studied seeds had moved <10 mm, 65.8% by <5 mm, and 21.7% by <2.5 mm. The maximum resulting change in volume of the prescription isodose line was <3%.

### Adverse Events

There were no severe acute radiation-related toxicities, with the exception of two patients who developed wound breakdown requiring local wound care. Of these two patients, one had delayed wound healing and developed contralateral neck and distant recurrence and died of disease afterward; the other developed cellulitis on the implanted neck 11 months after implantation and required surgical drainage. The second patient remained free from disease at last follow up, approximately 2 years after Cs-131 implant. Overall grade 1–2 acute toxicities attributable to radiotherapy were observed in 4 (33%) patients. Grade 3 acute toxicities were observed in 2 (16.7%) patients. The most common acute toxicities attributable to radiotherapy were wound infection and laryngeal edema. After surgery, patients remained hospitalized for a median of 6 (3–9) days.

### Disease Recurrence and Survival Outcomes

**Figure 3:**
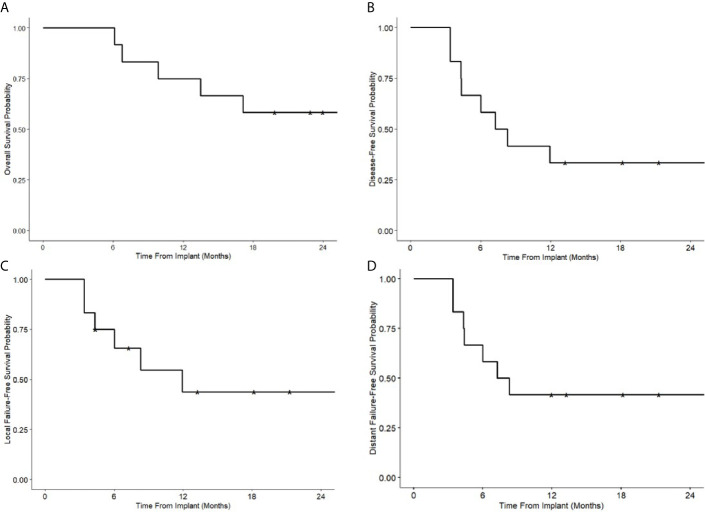
Kaplan-Meier survival curves following Cs-131 implantation. **(A)** Overall survival. **(B)** Disease-free survival. **(C)** Local failure free survival. **(D)** Distant failure free survival.

The median follow-up was 21.4 (6.1–40.8) months after implantation for all patients and 29.3 (19.8–40.8) months for patients who remain alive. The 1- and 2-year overall survival was 75 and 58%, respectively. Progression-free survival was 33%, local failure-free survival 44%, and distant failure-free survival 42% at both one and 2 years (**Figure 3**). At last follow up, eight (67%) patients had recurrences; three (25%) patients recurred local-regionally alone, three (25%) distant alone, and two (17%) with both -loco-regional and distant recurrences. Of the 5 (42%) with loco-regional recurrences, only 1 (8%) patient failed in-field. One patient failed at the field edge, one in the contralateral neck, one in the ipsilateral neck distant from the implant, and 1 patient who had implant in the neck for nodal recurrence of oropharyngeal cancer had a new primary laryngeal cancer. Five patients (42%) died. Four died with recurrence of their disease, and one died of other causes with no evidence of recurrence prior to death.

## Discussion

A growing body of evidence suggests that adjuvant re-irradiation in recurrent HNC improves local control, and may improve survival in selected cases (14–17). However, re-irradiation is often complicated by prior full dose irradiation, during which adjacent critical structures may receive their maximum tolerable dose with chronic alterations to their function. Several studies have reported rates of acute grade 3 and 4 toxicity after IMRT-based re-irradiation as high as 20–50% (8, 15, 17, 18). Rates of carotid blowout and mucocutaneous fistula, among the most serious complications, were reported between 2 and 5% in large series using IMRT (9). The use of more highly conformal techniques are attractive for re-irradiation in recurrent HNC in order to deliver sufficient high dose radiation while spare previously irradiated normal tissues.

Brachytherapy provides the possibility of optimal dose conformity, with sharp dose fall-off and no entrance/exit dose or low-dose bath to nearby normal tissues, leading to fewer side effects compared to EBRT (19). Brachytherapy performed at the time of surgery is convenient for patients, who would otherwise require 4–6 weeks of wound healing and 6–7 weeks of daily treatment with EBRT. Reported brachytherapy approaches largely utilize catheter-based high-dose rate (HDR) brachytherapy with an iridium-192 (Ir-192) isotope, as well as permanent implant low-dose rate (LDR) with iodine-125 and Cs-131 seeds. Several retrospective and prospective studies have reported feasibility of brachytherapy in postoperative re-irradiation in recurrent HNC (20–26), with comparable rates of survival and radiation-induced toxicity to our experience reported here.

HDR catheter-based brachytherapy allows for the precise tailoring of the dose distribution during pre-planning, with optimization of dwell times and positions, and image-guided catheter placement for each fraction. While these features allow for a theoretically superior dose-distribution compared to LDR brachytherapy, placement of the catheters can be challenging, especially in the surgically manipulated tissue. HDR techniques often require multi-fraction treatments which can be difficult for patients to endure. Despite these challenges, multiple reports on the use of HDR brachytherapy in the post-operative setting and as definitive therapy for unresectable recurrent HNC have been published, with local control rates at 2 years at approximately 60–70%, and rates of severe (grade 3 or higher) acute toxicities widely variable at approximately 10–50% (20–27). Irradiation of the flap and surgically manipulated tissues were generally well tolerated, with rates of grade 3 or higher wound complications typically 10% or less in reported series.

LDR brachytherapy following surgery in recurrent HNC has been used successfully for several decades. Permanent seed implant can be performed at the time of surgery and provides convenience and ease of use compared to HDR brachytherapy. Seeds typically come in strands, mesh, or can be individually placed, and can be customized to a particular patient’s surgical bed and at risk tissues, which may be distorted from their normal planes after surgical manipulation. Cs-131 is a relatively newer isotope with a higher energy at 30.4 kEV and shorter half-life at 9.7 days compared to iodine-125 and paladium-103 seeds, allowing for a higher biological effective dose (BED) and minimizing changes in the dose distribution due to seed migration or changes in the surrounding tissue. These features may increase the safety and quality of dose delivery and may also reduce toxicity.

Our institutional experience is consistent with previous reports using similar techniques. Pham et al. reported 18 patients with recurrent HNC treated with surgery and intraoperative placement of Cs-131 plaque to treat the tumor bed with an additional 5 mm margin to 80 Gy (28). Rates of grade 3 toxicity were similar to our experience regarding wound complication, with no grade 4 or 5 toxicities reported. There were no other severe acute or late radiation-related toxicities. They reported overall and progression-free survival at 18 months were 45 and 37% respectively, similar to our report at 58 and 33% respectively at 24 months. Bar-ad et al. reported 15 patients treated with Cs-131 brachytherapy for re-irradiation after surgical resection (29). The mean implant dose was 56.1 Gy, with similar treatment implementation to our approach, though no toxicity or tumor control endpoints were reported.

The overall local recurrence rate of 44% in our cohort is comparable to other series utilizing post-operative LDR brachytherapy with other isotopes (24, 30). The overall survival and recurrence free survival in our cohort is also comparable to a recently reported multi-institutional trial by Awan et al. of re-irradiation using IMRT to 60–66 Gy and concurrent cisplatin and cetuximab (14). They reported that the 1 year overall survival and recurrence free survival were 60.4 and 34.1%, respectively. However, there were significantly higher rates of grade 3 and 4 toxicity as would be expected with the use of concurrent systemic therapy. Of note, our patient population was high-risk, with a high median age of 74.5 years, medical comorbidities, and intensive prior therapy, all being strong competing risks for overall survival. Brachytherapy with Cs-131 did not prolong the hospital stay and the whole treatment period, which is an important consideration given the poor prognosis of this group of patients.

There are some limitations to consider for our study. Our cohort size is small with only 15 patients recruited from a single institution, and our study design is non-randomized, making generalizable conclusions difficult to formulate from our data alone. A larger study is needed to fully assess the toxicity and efficacy associated with brachytherapy in this setting. Our patients had considerable heterogeneity in the site of their primary cancer, with sites including the base of tongue, larynx, oral tongue, oropharynx, and skin, which carries some implications regarding tumor biology and risks from the initial therapy that cannot be fully known. The strengths of our study include the prospective collection of toxicity data, the consistency of implantation technique and evaluation, and concordance with other reported series employing similar techniques with regard to radiation safety parameters, toxicity, and outcomes.

Out of twelve patients in our study, only one had an in-field recurrence in the high dose region of the implant, indicating the feasibility of brachytherapy in achieving local control in the tumor bed in a presumably radioresistant recurrence. The majority of failures in our series occurred outside the treatment field; five patients recurred at distant sites, and two of three patients with local recurrences alone had out-of-field recurrences. This indicates the need for systemic treatment in combination with brachytherapy that not only addresses radioresistance in recurrent tumors, but the propensity for distant metastasis. Immunotherapy has been shown to have significant activity in the metastatic and recurrent HNC. The KEYNOTE-048 trial showed significantly improved overall and progression-free survival in patients with metastatic and recurrent HNC with either single agent pembrolizumab or combination of pembrolizumab and chemotherapy (31). The tolerability and efficacy of pembrolizumab in this setting make it an attractive candidate for therapeutic escalation in combination with brachytherapy. A new multi-center phase 1b/II trial combining PD1 inhibition using pembrolizumab and cesium-131 brachytherapy with salvage surgery to enhance immunogenicity and improve local control in head and neck cancer (ClinicalTrials.gov Identifier: NCT04340258) has been developed and will be activated with the hope of improving both local and distant disease control in recurrent head and neck cancer.

## Conclusions

It appears that Cs-131 implant after surgical resection in recurrent HNC is feasible and safe. There were no unexpected severe acute or late toxicities following the procedure. Most failures were out-of-field or distant failures; only one patient recurred in-field. Exploration of combination of immunotherapy and Cs-131 implant is warranted.

## Data Availability Statement

The raw data supporting the conclusions of this article will be made available by the authors upon request.

## Ethics Statement

This study involved human subjects and was reviewed and approved by the University Hospitals Institutional Review Board.

## Author Contributions

All authors discussed and conceived of the study design. MK, TP, ZZX, and MY performed data collection, analysis, and interpretation. MK performed the statistical analysis. All authors contributed to the article and approved the submitted version.

## Funding

The study was partly funded by IsoRay™ (Richland, WA).

## Conflict of Interest

The authors declare that the research was conducted in the absence of any commercial or financial relationships that could be construed as a potential conflict of interest.

## Abbreviations

HNC, head and neck cancer; EBRT, external beam radiation therapy; Cs, cesium; IMRT , intensity-modulated radiation therapy; SBRT, stereotactic body radiation therapy; OAR, organ at risk; CT, computed tomography; PET, positron emission tomography; HDR, high dose-rate; LDR, low dose-rate; NRC, Nuclear Regulatory Commission; IRB, institutional review board.
